# Measuring health facility readiness and its effects on severe malaria outcomes in Uganda

**DOI:** 10.1038/s41598-018-36249-8

**Published:** 2018-12-18

**Authors:** Julius Ssempiira, Ibrahim Kasirye, John Kissa, Betty Nambuusi, Eddie Mukooyo, Jimmy Opigo, Fredrick Makumbi, Simon Kasasa, Penelope Vounatsou

**Affiliations:** 1 Swiss Tropical and Public Health Institute, Basel, Switzerland; 2 University of Basel, Basel, Switzerland; 3 Makerere University School of Public Health, Kampala, Uganda; 4 Ministry of Health, Kampala, Uganda; 5 Makerere University Economic Policy Research Centre, Kampala, Uganda

## Abstract

There is paucity of evidence for the role of health service delivery to the malaria decline in Uganda We developed a methodology to quantify health facility readiness and assessed its role on severe malaria outcomes among lower-level facilities (HCIIIs and HCIIs) in the country. Malaria data was extracted from the Health Management Information System (HMIS). General service and malaria-specific readiness indicators were obtained from the 2013 Uganda service delivery indicator survey. Multiple correspondence analysis (MCA) was used to construct a composite facility readiness score based on multiple factorial axes. Geostatistical models assessed the effect of facility readiness on malaria deaths and severe cases. Malaria readiness was achieved in one-quarter of the facilities. The composite readiness score explained 48% and 46% of the variation in the original indicators compared to 23% and 27%, explained by the first axis alone for HCIIIs and HCIIs, respectively. Mortality rate was 64% (IRR = 0.36, 95% BCI: 0.14–0.61) and 68% (IRR = 0.32, 95% BCI: 0.12–0.54) lower in the medium and high compared to low readiness groups, respectively. A composite readiness index is more informative and consistent than the one based on the first MCA factorial axis. In Uganda, higher facility readiness is associated with a reduced risk of severe malaria outcomes.

## Introduction

The global malaria burden has declined in the last decade with the incidence of cases and malaria-related deaths reducing by 18% and 48%, respectively during 2000–2015^1^. Nevertheless, the disease remains a major public health problem and accounts for over 210 million cases and 420,000 deaths annually, affecting mainly the sub-Saharan Africa^2^.

In Uganda, malaria is a major leading cause of hospitalization and death, responsible for 30–50% of all health facility outpatient visits, 15–20% hospital admissions, and over 20% of hospital deaths^3^. Malaria burden has also reduced in the last few years with malaria incidence declining by over 75% between 2000 and 2015^3^. Although the contribution of control interventions towards malaria decline in Uganda has been investigated^4^, there is a paucity of evidence for the role health system strengthening has had on this success. This may be attributed mainly to the lack of direct measurements of health systems strengthening^5^, and partly to the weak routine data collection systems in developing countries^6^. The rollout of the District Health Information System version 2 (DHIS2) in Uganda has facilitated electronic reporting of routinely collected health facility data and has led to improvements in data quality^7^.

Health system strengthening can be measured indirectly using proxies of its six building blocks, that is, governance, health workforce, health financing, health technologies, health information and service delivery^8^. Service delivery is primarily concerned with immediate outputs of a national health system^9^. The proxy measure for service delivery is health facility readiness defined in terms of general service and service-specific readiness indicators^5^ estimated from health facility surveys.

General service readiness refers to the overall capacity of health facilities to provide health services and is measured by the availability of tracer items in five domains, namely; basic amenities, basic equipment, standard precautions for infection prevention, diagnostic capacity and essential medicines^10^. Service-specific readiness, on the other hand, refers to the capability of health facilities to provide a service of minimum acceptable standards, and is measured by the availability of the following tracer items necessary for the provision of a particular service; trained staff, service delivery guidelines, equipment, diagnostic capacity, medicines and commodities^10^.

Although measurements of facility readiness is crucial for health planning and decision making, the implementation of nationally representative facility surveys in Uganda has suffered from lack of funds. The most recent survey namely, the Uganda Service Delivery Indicator (USDI) was conducted in 2013 and it was supported by the World Bank^11^. USDI provides a set of metrics for benchmarking service delivery performance in health and education and assesses the quality of basic health services and of services related to primary education. It adopted health facility assessment tools used in service provision assessments designed by the World Health Organization (WHO)^12^. A high number of health facility readiness indicators, corresponding to tracer items can be generated from these surveys, each measuring a different attribute of readiness but no single indicator is sufficient to summarize all aspects of facility readiness. Therefore, a need arises to develop a single index of readiness that represents the vast array of readiness indicators characterizing health system functioning and its effect on health outcomes.

Facility readiness scores and categorical indices derived from score quantiles have been developed in assessment surveys conducted in several countries including Nigeria^13,14^, Ghana^15^, Haiti^16^, Tanzania^17^, Brazil^18^, Malawi and Nepal^19^, Kenya, Namibia and Rwanda^20^ to assess the effects of health facility readiness on health outcomes. In most of these studies the score was developed using Principal Component Analysis (PCA) designed for summarizing continuous variables^21^, despite the fact that the data collected from the facility assessments surveys are mainly binary in nature. Multiple correspondence analysis (MCA) is the most appropriate technique for this type of categorical data^15,22,23^. A few studies that have employed MCA to construct a facility readiness score used the first factorial axis to represent overall facility readiness^24,25^. However, the use of this single-axis score is unlikely to fulfill the Global First Axis Ordering Consistency (FAOC-G) property^26^ which means that the score monotonically increases/decreases for all indicators. The FAOC-G property ensures that the absence of any readiness indicator from a facility will contribute to a lower readiness score than its presence. Failure of the FAOC-G will result to inconsistent and meaningless readiness score. Asselin (2009)^26^ proposed a composite index based on more than one MCA axis to remedy the construction of inconsistent poverty scores. To our knowledge, composite MCA scores have not been used in constructing indices measuring health systems-related performance. More so, construction of readiness scores in the above studies did not preselect indicators that were most relevant for the outcome(s) of interest. Variable selection formulated in a geostatistical modeling framework has been shown to identify covariates that explain most variation in the outcome^27^. It is expected that a readiness score obtained from indicators most relevant for the health outcome would be more informative.

In this study, we linked the USDI survey of 2013 with data on severe malaria (deaths and severe cases) data reported in the Health Management Information System (HMIS) to assess the effects of facility readiness on severe malaria outcomes. A composite readiness score was created by exploiting more than one factorial axis of the MCA of the most relevant general service and malaria specific readiness indicators identified through geostatistical variable selection. Results from this study provide methodology on constructing facility readiness indices and provide information to the Ministry of Health (MoH) and other stakeholders on the overall readiness of lower level health facilities in Uganda to deliver malaria services, and the role of this effect on the risk of severe malaria outcomes.

## Methods

### Settings

Uganda is located in the SSA region and ranks among the top 15 countries that contribute to 90% of the global malaria burden. Malaria transmission is stable and perennial in 95% of the country, but the entire population is at risk^28^. The remaining 5% of the country comprises of unstable and epidemic-prone transmission areas situated in highlands of the south-western, and areas around the mountains Rwenzori in the mid-western region and Elgon in mid-eastern. *Plasmodium falciparum* is the dominant parasite species and the most dangerous with the highest case-fatality rate. The primary vector is *Anopheles gambiae s.l*. which breeds in temporary stagnant water, while An*. funestus* is the second most important vector and breeds mainly in permanent water bodies.

### National health system

The health system in Uganda is decentralized with the Ministry of Health responsible for policy formulation, quality assurance, resource mobilization, capacity development, technical support, and provision of nationally coordinated services such as epidemic control, coordination of health research and monitoring and evaluation of overall sector performance. Health care services are delivered through a tiered structure of facilities consisting of hospitals and Health Centers (HC) IV, HCIII, HCII and HCI at district, Health Sub-District (HSD), sub-county, parish and village levels, respectively^29^. Hospitals are further classified into district, regional referral, national referral serving district, region and country-level populations. The HCI is the lowest level and first point of contact. It is headed by village health teams (VHT)/community medicine distributors who are largely volunteers, targeting smaller populations of 1000 people.

### Data sources

#### Severe malaria outcomes

Data on severe malaria outcomes was extracted from the Health Management Information System (HMIS) for the period January–December 2013. Two severe malaria outcomes were defined, namely, the cumulative number i) of malaria deaths and ii) of severe malaria cases leading to hospitalization during 2013. Both outcomes were considered for the analyses of HCIII data, but only the latter for HCIIs due to the limited scope as diagnosed severe cases are referred to HCIIIs and other higher level facilities.

#### Statistical methods

Data from the USDI survey were used to construct readiness indicators following standard definitions^10^. In particular, we created (i) general service readiness indicators for the five domains (i.e. basic amenities, basic equipment; standard precautions for infection prevention; diagnostic capacity and essential medicines) and (ii) malaria-specific indicators. Readiness indicators were defined as binary variables, taking the value ‘1’ if the tracer item was available at the facility and ‘0’ otherwise. Availability and functionality of items were confirmed through direct observation by the interviewer prior to data recording in the questionnaire. Furthermore, domain readiness indicators for each of the five domains of the general service readiness and for the domain of malaria services were defined as availability of all tracer items that belong to a particular domain. A facility was assigned 1 if all tracer items constituting a domain were found at the facility and 0 otherwise.

Bayesian geostatistical negative binomial models using stochastic search variable selection were fitted to the severe malaria outcomes to select the most important facility readiness indicators. For each readiness indicator, a Bernoulli variable was introduced with Bernoulli probability corresponding to the inclusion of the indicator in the model (details are provided in the Appendix). Spatial correlation was taken into account by assuming a Gaussian process on health facility locational random effects. The models were fitted separately on severe malaria and malaria mortality for HCIII facilities and on severe malaria for HCII facilities.

MCA was applied to the most important *K* readiness indicators, ${X}^{k},\,\,k=1,\,\ldots ,\,K$ selected with posterior inclusion probabilities of at least 50% to construct a facility readiness score. For each indicator *X*^*K*^, two binary variables were created, ${X}_{0,i}^{k}$ and ${X}_{1,i}^{k}$ corresponding to the presence and absence of the indicator/tracer from the facility, respectively. In particular, ${X}_{0,i}^{k}$ takes the value 1 when the tracer *k* is absent from facility *i* (i.e. *X*^*K*^ = 0) and 0 otherwise. Similarly, ${X}_{1,i}^{k}$ takes the value 1 when the tracer *k* is present in facility *i* (i.e. *X*^*K*^ = 1) and 0 otherwise. A readiness score ${F}_{i}^{a}$ for health facility *i*, based on the *a*^*th*^ factorial axis of MCA was defined by ${F}_{i}^{a}=\frac{1}{K}\sum _{k=1}^{K}\,\sum _{{j}_{k}=0}^{1}\,{W}_{{j}_{k}}^{a,k}{X}_{{j}_{k},i}^{k}$, where *j*_*k*_ indicates the value of *X*^*K*^ and the weights ${W}_{{j}_{k}}^{a,k}$ are the column standard coordinates on the *a*^*th*^ factorial axis corresponding to ${X}_{{j}_{k},i}^{k}$. Typically, a score is defined on the first factorial axis, i.e., *a* = 1. Following the approach proposed by Asselin (2009), we defined the composite readiness score *F*_*i*_ by ${F}_{i}=\frac{1}{K}\,\sum _{k=1}^{K}\,\sum _{{j}_{k}\in \{0,1\}}\,\sum _{a=1}^{L}\,\delta (k-a){W}_{{j}_{k}}^{a,k}{X}_{{j}_{k},i}^{k}$, where *L* is the number of factorial axes used in the composite score and $\delta (k-a)$ is the Dirac delta function which takes the value 1 when the weights related to ${X}_{{j}_{k},i}^{k}$ are selected from from the *a*^*th*^ factorial axis and 0 otherwise, that is, $\delta (k-a)=1$ if *k* = *a* and $\delta (k-a)$ = 0 if $k\ne a$. Identification of the factorial axis that will represent the *X*^*K*^ indicator depends on a discrimination measure calculated for each indicator and axis, measuring the contribution of the indicator to the total variance explained by the axis. To improve interpretation of the score we translated the weights so that the absence category (*j*_*k*_ = 0) of the *X*^*K*^ indicator received a zero weight and the presence one (*j*_*k*_ = 1) received a strictly positive weight representing the gain in the readiness increase measured by the axis *a* when a facility *i* acquires the *kth* tracer. Therefore, the ${W}_{{j}_{k}}^{a,k}$ in *F*_*i*_ is replaced by ${W}_{{j}_{k}}^{+a,k}$ where ${W}_{0}^{+a,k}$ = 0 and ${W}_{1}^{+a,k}$ = ${W}_{1}^{a,k}$ − ${W}_{0}^{a,k}$. Details on this procedure are provided in the Appendix.

A separate composite score was derived for each health facility level due to differences in mandate and service scope across levels. A readiness index was created from the readiness score as a categorical variable with three levels for both HCIIIs and HCIIs based on the tertiles of the distribution of the composite score.

Descriptive statistics, that is, frequencies, proportions and chi-square tests were used to summarize and compare readiness indicators and the index by facility level and other health facility characteristics. Geostatistical Bayesian negative binomial models were fitted separately by facility level to assess the effect of health facility readiness on the severe malaria outcomes. The models were adjusted for facility location (rural/urban), management authority (government/private) and distance to district headquarters.

Descriptive analysis and MCA were conducted in STATA^30^ and Bayesian models were fitted in OpenBUGS^31^ using Markov Chain Monte Carlo (MCMC) simulation. Parameters were summarized by their posterior medians and 95% Bayesian Credible intervals (BCIs). Modeling details are provided in the Appendix.

## Results

### Health facility characteristics

**Figure 1:**
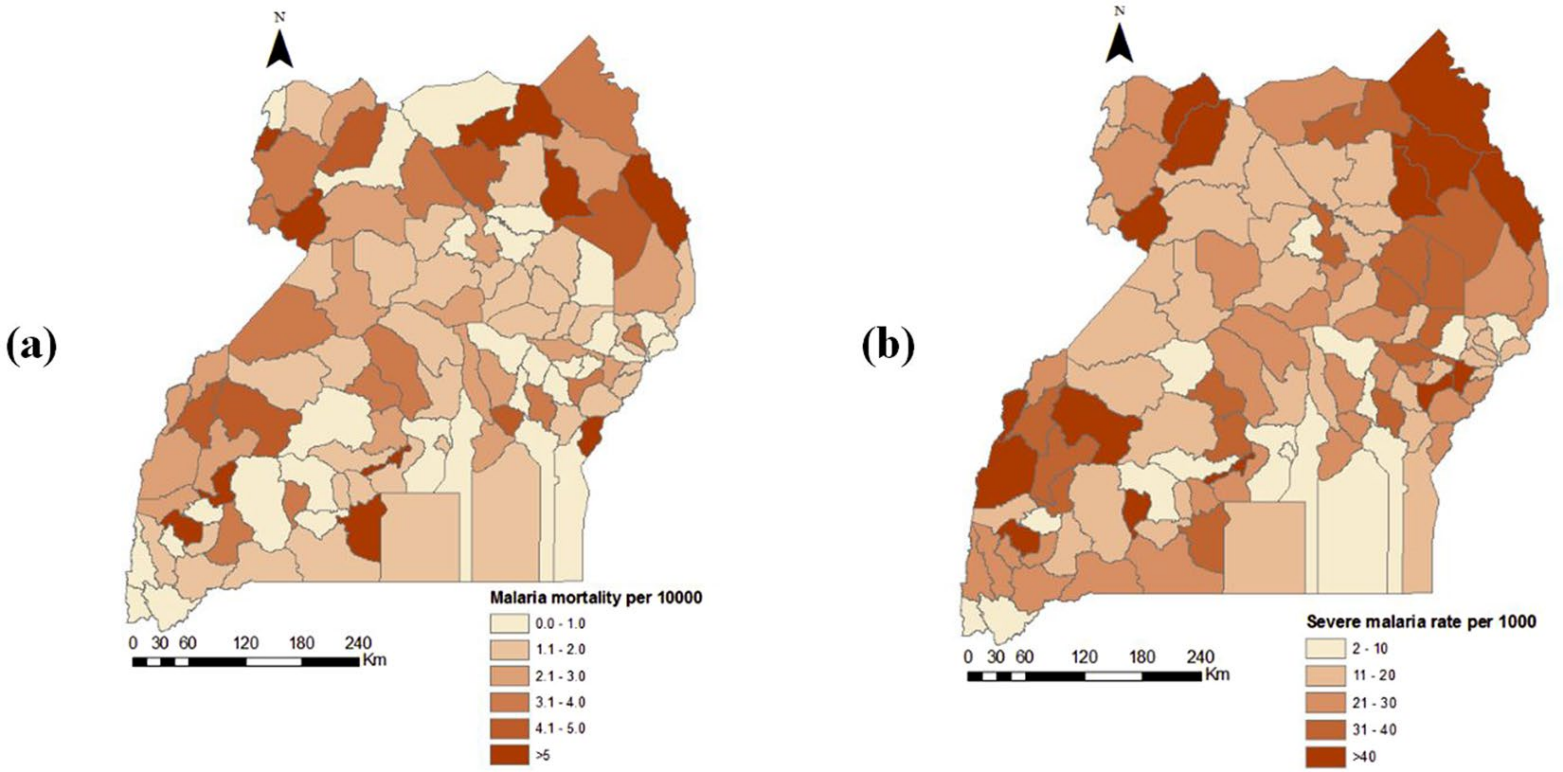
Geographical distribution of severe malaria outcomes in Uganda in 2013; (**a**) mortality, (**b**) severe cases.

A total of 250 health facilities participated in the health facility assessment survey but only 207 (82.8%) reported in the HMIS consistent and complete data on severe malaria outcomes during January–December 2013. Six out of the 207 were higher level facilities (i.e. hospitals and HCIVs) and were excluded due to insufficient sample size. The characteristics of the 201 facilities included in the analysis are presented in Table 1. Most facilities were HCIIIs, government-managed, rural-based, and were located more than 10 km from district headquarters. The average travel time from the district headquarters to a facility using public means of transport was an hour. HCIIIs offered outpatient consultations on average seven days a week, 15 hours a day. HCIIs operated six days a week, 12 hours per day. A total of 87,719 severe malaria outcomes were reported from the 201 facilities during the study period, 86,848 (99%), of which were severe malaria cases and 871 were malaria-related deaths. The majority (61,642) of outcomes were reported by HCIIIs. The number of severe malaria cases and malaria-related deaths was twice as high in children less than 5 years than in older individuals. The distribution of severe malaria outcomes is shown in Fig. 1 and suggests a higher burden in areas of the north and western parts of the country compared to the central areas.

**Table 1:** Health facility characteristics.

Characteristic	Total (N = 201) n (%)	HCIIIs (N = 105) n (%)	HCIIs (N = 96) n (%)
**Managing authority**			
Government	146 (72.6)	76 (72.4)	71 (74.0)
Non-government	55 (27.4)	29 (27.6)	25 (26.0)
**Location type**			
Rural	166 (82.6)	83 (79.1)	83 (86.5)
Urban	35 (17.4)	22 (21.0)	13 (13.5)
**Distance to district headquarters**			
0–10 km	52 (25.9)	28 (26.7)	24 (25.0)
>10 km	149 (74.1)	77 (73.3)	72 (75.0)
**Region**			
Central	47 (23.4)	23 (21.9)	24 (25.0)
Eastern	51 (25.4)	29 (27.6)	22 (22.9)
Kampala	10 (5.0)	5 (4.8)	5 (5.2)
Northern	33 (16.4)	22 (21.0)	11 (11.5)
Western	60 (29.9)	26 (24.8)	34 (35.4)
	**Mean (sd)**	**Mean (sd)**	**Mean (sd)**
Days per week facility is open	6.4 (1.0)	6.7 (0.9)	6.0 (1.1)
Hours per day facility is open	12.9 (6.4)	14.1 (6.9)	11.6 (5.5)
Travel time from facility to district headquarters (hours)	1.1 (1.1)	1.0 (1.1)	1.2 (0.9)
Proportion of malaria deaths*	%	%	%
All ages	0.98	1.14	0.61
<5 years	1.09	1.13	0.96
>=5 years	0.85	1.16	0.31

^*^Of the total severe malaria cases.

### General service and malaria specific readiness indicators

General service and malaria specific readiness indicators for HCIIIs and HCIIs are presented in Table 2 by domain along with their posterior inclusion probabilities obtained from the geostatistical variable selection.

**Table 2:** Posterior inclusion probabilities estimated from Bayesian geostatistical variable selection and frequency distribution of general service and malaria specific readiness indicators.

Readiness indicator	HCIIIs N = 105			HCIIs N = 96	
	Readiness n (%)	Posterior inclusion probabilities		Readiness n (%)	Posterior inclusion probabilities
General service		Severe malaria cases (%)	Malaria deaths (%)		Severe malaria cases (%)
**Basic amenities** ^†^	**3 (2.9)**			**0 (0.0)**	
Uninterrupted power supply	45 (42.9)	47.0	37.3	32 (33.3)	34.1
Improved water source inside or within source of facility	37 (35.2)	68.0*	67.8*	21 (21.9)	44.0
Access to adequate sanitation facilities for clients	94 (89.5)	42.0	44.3	88 (91.7)	43.6
Communication equipment (phone or short wave radio)	22 (21.0)	41.3	43.4	6 (6.3)	43.0
Access to computer with email/internet access	21 (20.0)	38.7	37.9	8 (8.3)	43.0
Emergency transportation	16 (15.2)	34.4	39.8	5 (5.2)	60.8*
**Basic equipment** ^†^	**63 (60.0)**			**38 (39.6)**	
Adult scale	87 (82.9)	61.1*	59.2*	70 (72.9)	34.4
Child scale	89 (84.8)	38.8	39.2	70 (72.9)	60.6*
Thermometer	88 (83.8)	42.4	42.6	75 (78.1)	56.5*
Stethoscope	98 (93.3)	39.7	44.1	80 (83.3)	32.4
Blood pressure apparatus	91 (86.7)	42.6	39.4	77 (80.2)	33.2
**Standard precautions for infection prevention** ^†^	**5 (4.8)**			**4 (4.2)**	
Sterilization equipment	29 (27.6)	36.3	39.3	7 (7.3)	40.4
Appropriate storage of sharps waste	101 (96.2)	40.9	42.2	93 (96.9)	75.7*
Safe final disposal of sharps	15 (14.3)	39.1	40.9	10 (10.4)	42.6
Disposable syringes with disposable needles	101 (96.2)	46.9	40.5	93 (96.9)	50.7*
Disposable gloves	98 (93.3)	55.6*	64.0*	94 (97.9)	51.6*
**Diagnostic capacity** ^†^	**34 (32.4)**			**6 (6.3)**	
Malaria RDTs	83 (79.1)	70.5*	72.8*	72 (75.0)	38.0
Blood glucose	52 (49.5)	39.2	27.2	12 (12.5)	57.0*
HIV diagnostic capacity	89 (84.8)	47.7	51.0*	37 (38.5)	30.3
Urine dipstick	74 (70.5)	25.8	39.5	14 (14.6)	40.0
**Essential medicines** ^†^	**5 (4.8)**			**0 (0.0)**	
Amoxicillin syrup/suspension or dispersible tablet	24 (22.9)	45.0	50.7*	17 (17.7)	61.0*
Ampicillin powder for injection	72 (68.6)	50.5*	53.2*	7 (7.3)	45.1
Ceftriaxone injection	41 (39.1)	63.5*	56.0*	60 (62.5)	32.5
Gentamicin injection	52 (49.5)	52.2*	56.2*	21 (21.9)	38.2
Magnesium sulphate injectable	58 (55.2)	55.9*	58.6*	5 (5.2)	46.4
Oral rehydration solution	87 (82.9)	31.9	39.2	74 (77.1)	38.0
Oxytocin injection	58 (55.2)	57.3*	53.3*	5 (5.2)	41.4
**Zinc sulphate tablets, dispersible**					
tablets or syrup	77 (73.3)	62.9*	54.7*	64 (66.7)	41.4
**Malaria service** ^†^	**45 (42.9)**			**8 (8.3)**	
Microscopy	81(77.1)	63.8*	65.5*	16 (16.7)	74.2*
Artemisinin Combination Therapies (ACTs)	88 (83.8)	38.4	38.0	86 (89.6)	39.3
Fancidar	94 (89.5)	34.6	43.8	77 (80.2	28.6
Artesunate	5 (4.8)	45.4	41.7	2 (2.1)	63.9*

^†^Domain readiness indicators are defined as availability of all tracer items belonging to the domain.

*Indicators with posterior inclusion probabilities of >50% were included in the construction of the facility readiness score.

Results show that basic amenities readiness was achieved in only three HCIII facilities and none in HCII. Access to adequate sanitation and availability of emergency transport were the most and least available tracer items in this domain. Urban-based facilities had a significantly higher basic amenities readiness compared to rural facilities (p-value = 0.023) (Table A1, Appendix).

Fifty percent of facilities (irrespective of level, HCIII and HCII) achieved basic equipment readiness. This readiness was significantly higher in HCIIIs, urban-located, private managed and in Central region facilities but did not differ by the proximity of a facility to district headquarters (Table A1, Appendix).

Standard precautions readiness was attained in close to five percent of the facilities, despite of high availability of most of the single tracer items. The commonest standard precaution items found at facilities were disposable syringes and needles, sharps container box, and disposable gloves, while the least available item was incinerator for final disposal of sharps. Standard precautions readiness was significantly higher among private managed (p-value = 0.007) and urban facilities (p-value = 0.002).

Diagnostic capacity readiness was met in only one-fifth of facilities. This readiness was more than five times higher in HCIIIs compared to HCIIs, two times more in urban than rural facilities. Diagnostics readiness was higher in private-managed facilities and highest in the Northern region but did not differ by the distance to district headquarters (Table A1, Appendix). The majority of the facilities had malaria RDTs but very few had urine dipstick used in measuring glucose levels. An average of three diagnostic tests were available in HCIIIs but only one in HCIIs.

Facility readiness for essential medicines was achieved in less than five percent in HCIII and in none of HCII facilities. On average, only three out of nine medicines assessed were available at both types of facilities. Availability of individual essential medicines was significantly higher in HCIIIs. Oral rehydration solution and zinc sulphate tablets were among the most available medicines, whereas magnesium sulphate and oxytocin injections were the least available. Private facilities, situated in urban places and close to the district headquarters had a significantly higher readiness for essential medicines.

Malaria-specific readiness was achieved in only one quarter of the facilities. It was eight times higher in HCIIIs and two times more in private managed compared to HCIIs and government managed facilities, respectively. However, readiness did not differ by location, region, and distance from district headquarters (Table A1, Appendix). In spite of the overall low malaria readiness, the proportion of facilities with RDTs and ACTs was high but varied with regions.

Geostatistical variable selection results showed that the same indicators were equally important for explaining variation in severe malaria cases and malaria-related deaths for HCIII facilities. More so, for HCIIIs, the essential medicines domain and for HCIIs the standard precautions for the infection prevention domain had the highest number of indicators related to malaria outcomes. Availability of RDTs, and appropriate storage of sharps waste were statistically important for HCIIIs and HCIIs, respectively. The disposable gloves were the only indicator selected in both HCIIIs and HCIIs types of facilities.

### Facility readiness score and index

MCA was applied on the readiness indicators selected from the variable selection procedure to obtain a readiness score. Bayesian variable selection model identified the same set of indicators in HCIIIs as being important for both severe malaria outcomes, therefore a single readiness score was created at this level.

**Figure 2:**
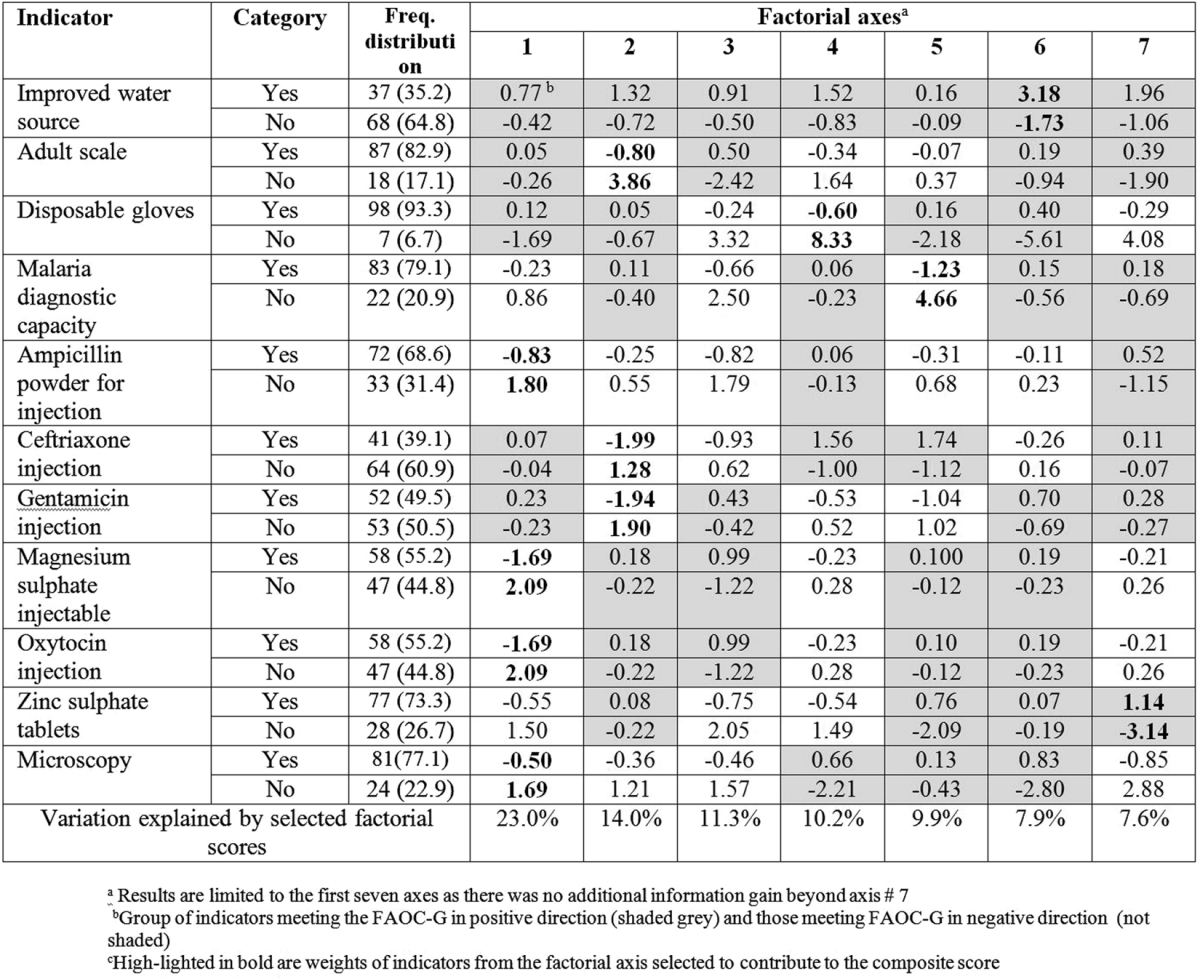
Standard coordinates of readiness indicators on the first seven factorial axes (HCIIIs).

**Figure 3:**
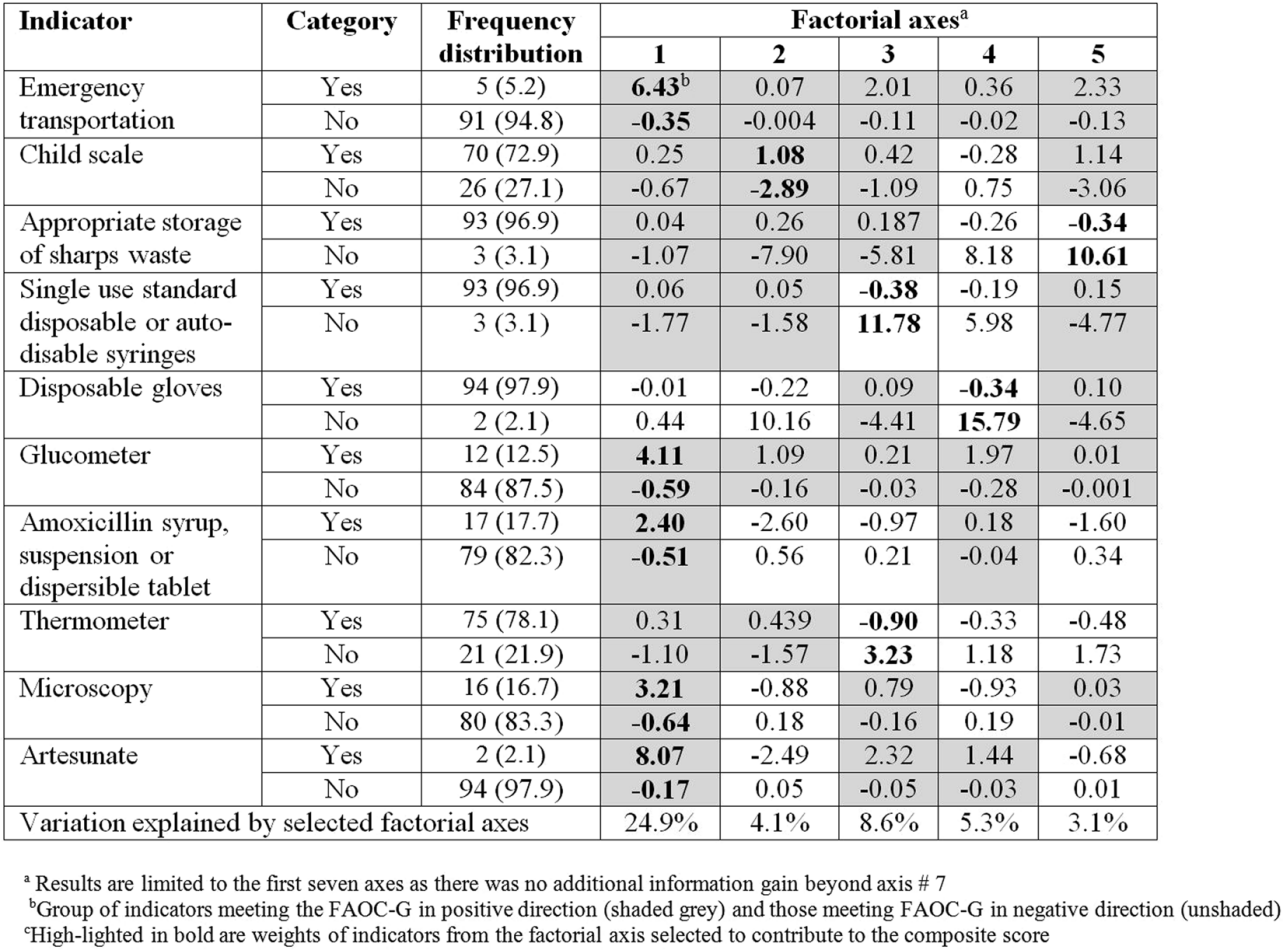
Standard coordinates of readiness indicators on the first five factorial axes (HCIIs).

Figures 2 and 3 display the standard coordinates of readiness indicators obtained from the first seven and five factorial axes for HCIIIs and HCIIs, respectively. Results show that for HCIIIs on the first factorial axis, a subset of five indicators met the FAOC-G requirement in the positive direction, while a second subset of six indicators met this requirement in the negative direction. Therefore, there are two subsets of indicators that are inconsistent and one subset should have been discarded, leading to a loss of information if we had constructed the score using the first factorial axis. For HCIIs, all but one indicator met the FAOC-G requirement. However, four of the selected indicators possess higher discrimination power on axes other than the first one.

The composite facility readiness score explained 47.6% of the total variation in the indicators from HCIIIs compared to 23% explained by the score based on the first factorial axes (Fig. A1, Appendix). Similarly, for HCIIs, the variation explained by the composite score was 45.8% which is almost two times higher than that explained by the first axis, i.e., 26.6%. Furthermore, our approach of including in the score construction the indicators identified by the variable selection gave a more informative score than the score we would have constructed from all indicators. In particular, the latter for HCIII explained 27.9% (composite) and 12.2% (first factorial axis) of the total variation. For HCII, these figures were 26.8% and 16.6%, respectively. Therefore, we used in the analysis the composite score based on the subset of selected indicators.

The indicators with the highest weights in the composite score (Tables A2 and A3 in the Appendix) are availability of disposable gloves and malaria Rapid Diagnostic Test (RDTs) kits (for HCIIIs), availability of disposable gloves, single use auto-disable syringes, and appropriate storage of sharps waste (for HCIIs). The composite scores show a normal distribution and a right-skewed distribution for HCIIIs and HCIIs, respectively (Fig. A2, Appendix).

**Figure 4:**
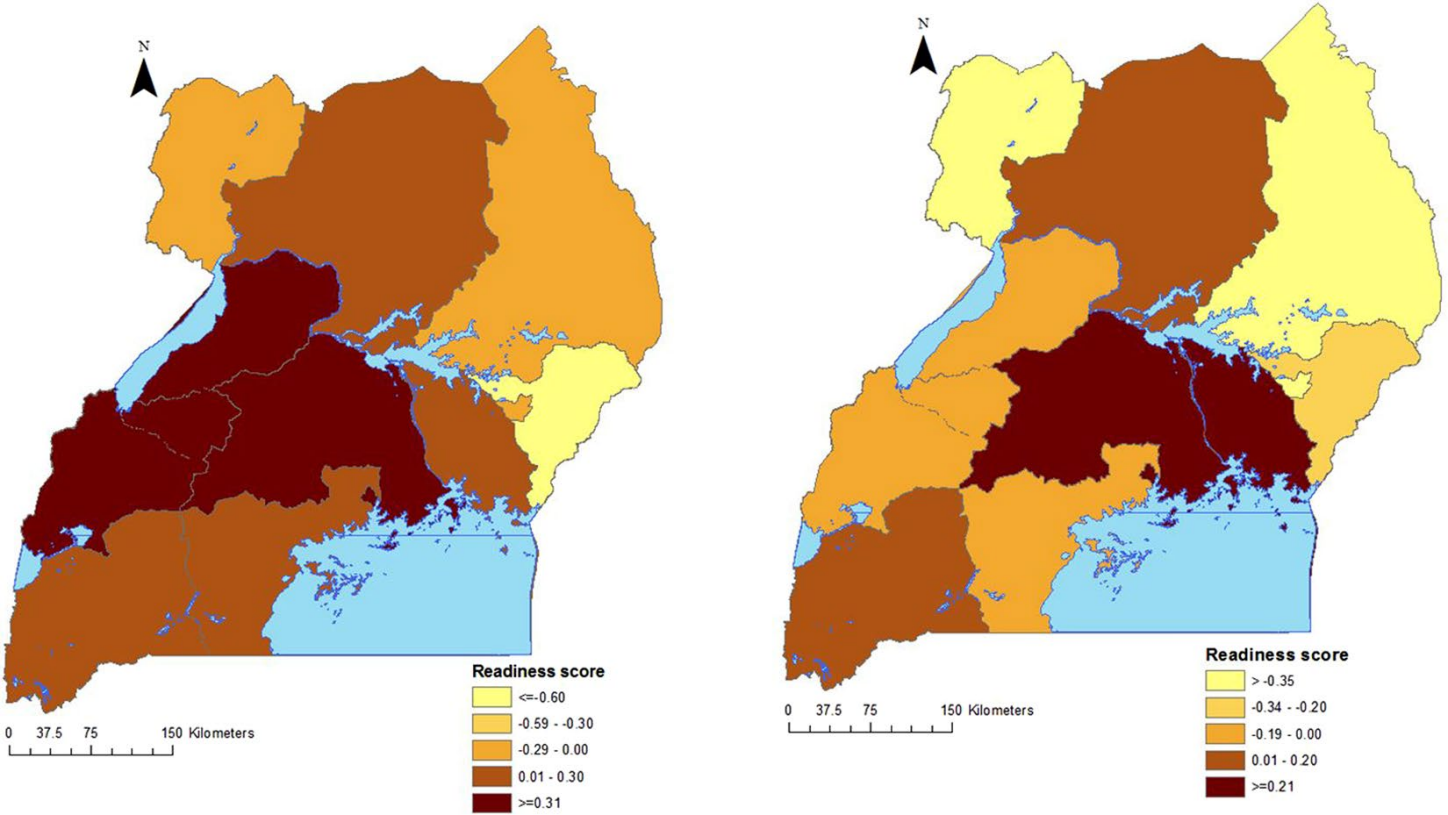
Regional distribution of facility readiness score; (**a**) HCIIIs, (**b**) HCIIs.

The regional average facility readiness score was higher in the central and southern located regions and lower in the eastern and northern areas of the country for both HCIIIs and HCIs (Fig. 4).

We used the tertiles for the score distributions to create a categorical readiness index with three categories for HCIIIs and HCIIs. The levels of the index ordered were treated as proxies for the low, medium and high readiness levels, respectively.

### Effects of facility readiness on severe malaria outcomes

Estimates of the effect of the composite facility readiness index on the malaria outcomes based on the selected indicators are presented in Table 3. For HCIIIs, malaria-related mortality decreased with increasing readiness. Mortality rate was 64% (IRR = 0.36, 95%BCI: 0.14–0.61) and 68% (IRR = 0.32, 95%BCI: 0.12–0.54) lower in the medium and high compared to low readiness groups, respectively. Malaria mortality was statistically lower in facilities located in urban areas, but did not differ by ownership, and distance of the facility to district headquarters. The incidence rate of severe malaria cases was 19% (IRR = 0.81, 0.56–0.93) and 76% (IRR = 0.24, 0.16–0.38) lower in the medium and high readiness groups, respectively compared to the low group. Severe malaria cases differed by facility location, but they were not related to district headquarters, and to ownership type (i.e. private vs government).

**Table 3:** Posterior estimates (median and 95% BCI) of the effects of composite facility readiness index on severe malaria outcomes estimated from Bayesian geostatistical negative binomial models.

Characteristic	HCIIIs		HCIIs
	Malaria deaths	Severe malaria cases	Severe malaria cases
	IRR (95%BCI)^a^	IRR (95%BCI)	IRR (95%BCI)
**Readiness index**			
Low	1	1	1
Medium	0.36 (0.14, 0.61)*	0.81 (0.56, 0.93)*	0.56 (0.26, 0.91)*
High	0.32 (0.12, 0.54)*	0.24 (0.16, 0.38)*	0.70 (0.42, 0.94)*
**Location**			
Rural	1	1	1
Urban	0.58 (0.20, 0.86)*	0.74 (0.63, 0.85)*	3.42 (0.92, 5.26)
**Ownership**			
Government	1	1	1
Private	0.76 (0.48, 1.90)	4.60 (0.90, 7.46)	1.34 (0.82, 3.04)
**Distance to district headquarters**			
<=10 km	1	1	1
>10 km	0.76 (0.48, 0.92)*	0.45 (0.36, 0.75)*	2.27 (1.34, 4.04)
**Spatial parameters**			
Spatial variance	1.45 (1.10, 1.82)	0.61 (0.49, 0.99)	0.58 (0.36, 0.71)
Range (km)	5.47 (2.77, 16.64)	4.26 (2.73, 13.21)	35.51 (4.65, 70.31)

*Statistically important effect; ^a^IRR: Incidence Rate Ratio.

For HCIIs, the incidence rate of severe malaria cases was 44% (IRR = 0.56, 0.26–0.91) and 30% (IRR = 0.70, 0.42–0.94) lower in the medium and high groups that the low one, respectively. The incidence of severe cases was twice as high among distant HCIIs compared to those near to the district headquarters, however, the distance effect was not important for HCIIIs. Geographical variation in severe malaria cases was higher in HCIIs than in HCIIIs.

We repeated the analysis of the relation between facility readiness and severe malaria outcomes using a composite readiness score constructed from all indicators to assess the impact of our approach on the estimated facility readiness effects. Results showed that the composite score constructed from all indicators suggested that the relation between readiness with severe malaria (for HCIIs) and with malaria deaths (for HCIIIs) were not statistically important (Table A4 in the Appendix).

## Discussion

We propose methodology to construct a composite facility readiness index for HCIIIs and HCIIs analyzing the Uganda service delivery indicators survey data of 2013 and used the derived index to assess the effects of health facility readiness on severe malaria cases and malaria-related deaths in the country during January-December 2013. The index was obtained by applying MCA based on the most relevant general service and malaria service readiness indicators for severe malaria outcomes identified through geostatistical variable selection.

Our findings suggest that the composite readiness score constructed from multiple factorial axes explains a higher proportion of the variation in the original data for both HCIIIs and HCIIs and therefore it is more informative than the one derived from the first axis. These findings are in agreement with results reported in economics literature in which the concept of composite score was first developed to evaluate poverty reduction programs^32–34^. However, the inclusion of multiple factorial axes in the score construction has not been applied yet in studies measuring health systems. These studies rather use the first MCA axis without any consideration of the Global Facility Axis Ordering Consistency (FAOC-G) property obtaining scores which are likely inconsistent not capable of describing all facets of readiness in the population of interest.

More so, the score based on the subset of indicators identified through variable selection contained more information and had an important effect on the risk of severe outcomes compared to the index created from all indicators. The probable explanation to this finding is that variable selection helps to weed out indicators from the index construction that have little or negligible relation to the health outcome of interest and hence resulting into a meaningful measure. Our study is the first to apply an objective procedure that selects the most important facility readiness indicators and a malaria-related facility readiness index.

Facility readiness is unevenly distributed across regions in Uganda with the northern regions having the least readiness compared to the central and southern located regions. These regional differences between the north and south may be explained by the recent war in the north that affected the health infrastructure and the availability and access of health services in this region^4,35^.

Indicators contributing the most weight to the composite index were those with a high coverage. These results are in agreement with findings from other studies^15,17,36–38^.

The readiness indicators that explained most variation in severe malaria outcomes differed between HCIIIs and HCIIs. This could be attributed to the different mandates of facilities at different levels owing to variations in service scope, staffing levels, infrastructure and equipment^39^.

The readiness score had a nearly normal distribution for HCIIIs and a right-skewed distribution for HCIIs. This is an indication of higher heterogeneity in readiness of HCIIs compared to HCIIIs and can be explained by the HCIIs’ limited capacity to provide quality basic healthcare services as a result of low staffing levels, high drug stock-outs, insufficient infrastructure, poor coordination and limited supervision unlike in HCIIIs or higher level facilities^39^.

Readiness of lower level facilities to provide malaria services was low despite the high availability of the domain-specific tracer items. Absence of microscopy diagnostic testing is the main reason for this shortcoming. The inadequate malaria readiness at the lower level facilities which serve a big proportion of the rural population may explain why the disease remains the leading cause of mortality in the country^29^. However, generally malaria-specific readiness was higher in HCIIIs, urban-located privately-managed facilities, and in facilities located nearer district headquarters. This is because HCIIIs receive more Primary Health Care (PHC) funding, have better infrastructure, more qualified personnel and are subject to more supervision from both technical and political teams at district and health sub-district level compared to HCIIs^29^.

The higher malaria readiness in privately managed facilities may be attributed to better medical equipment, well-maintained infrastructure, higher staffing levels, reduced staff absenteeism and higher supervision compared government-managed facilities^40^. Urban facilities have also higher malaria readiness most likely due to greater access to infrastructure including road network, national power grid and other public services, which eases transportation and delivery of commodities such as drugs, supervision, and improves staff morale boosting retention.

Facility readiness was very low for all general service domains with the exception of basic equipment. This can be related to inadequate government health sector funding which stands at 9.6% of the national budget and it is way below the Abuja Declaration target of 15%^41^. The low sector funding affects negatively the maintenance of infrastructure, causes stock-outs of essential drugs, slows down recruitment and motivation of the health workforce^29^. Readiness in the basic equipment domain was high most likely due to the durable nature and low cost of the tracer items that constitute this domain compared to other domains whose items may cost higher and require substantial massive capital investment or they are consumables such as drugs that require constant replenishment.

Availability of malaria RDTs was high despite of the very low diagnostic capacity readiness. This can be attributed to the country’s adoption of the WHO ‘Test and Treat’ campaign where free RDTs are provided to public and private facilities by MoH with support from Roll Back Malaria (RBM) partnership^42^. The finding also indicates that the majority of malaria cases reported in the HMIS are confirmed. Availability of glucometers for measuring blood glucose was low especially at HCIIs indicating a major setback in lieu of emerging evidence of a growing non-communicable diseases burden in Uganda^43^. More so, essential medicines readiness was low despite some medicines such as oral rehydration solution and zinc sulphate drugs were highly available. This result is consistent with MoH reports that highlight drug stock-outs as one of the major constraints to good service delivery in the country^44^.

Readiness of both HCIIIs and HCIIs was associated with a decline in malaria-related mortality and severe morbidity. These results underscore the significance of health facility performance and health systems strengthening in general on health outcomes.

A higher number of malaria deaths and severe cases was obtained among children less than 5 years. This is expected in countries with a stable and intense *P. falciparum* transmission^45^ where severe malaria manifests mostly in young children with less developed immunity, but becomes less common in older children and adults as acquired immunity gives increasing protection^46^.

A limitation of the study is that health facility records underestimate morbidity and mortality in developing countries because most people who fall sick don’t seek health care and a number of them die at home. Results from the 2014-15 malaria indicator survey reported only 80% of children less than 5 years old who had a fever sought care and treatment from a formal health facility^42^. This proportion is likely to be even higher among adults since treatment seeking is higher among children compared to adults, therefore a large proportion of severe malaria illnesses and deaths occur in people’s homes without coming to the attention of a formal health service^2^. Our findings are generalizable only for lower level facilities in Uganda namely, HCIIIs and HCIIs, and not for HCIVs and hospitals which serve as referral centers for lower facilities. Furthermore, our estimates for general service and malaria-specific readiness indicators may be overestimated since data on the availability of training guidelines and manuals was not collected in the survey.

## Conclusion

The composite readiness score created by exploiting more than one MCA factorial axis produces a more informative (explains more variation in the original data) and consistent health facility readiness measure that is capable of capturing all aspects of readiness unlike the index based on only the first axis. Higher facility readiness is associated with a reduced risk of severe malaria outcomes in the lower level facilities in Uganda. However, facility readiness to provide malaria treatment services is low. The biggest obstacle hindering lower level health facility readiness is the severe absence of basic amenities and stock-out of essential medicines. If the health facility readiness remains as it is now, the decline of severe malaria burden may be reversed, which will compromise the achievement of the goals of the Health Sector Strategic and Investment Plan development plan (HSSP) of 2015/16–2019/2020. The government should address lower level facility readiness gaps by increasing health sector funding to the levels recommended by Abuja declaration in order to achieve and sustain a substantial reduction in severe malaria burden in the country.

## Electronic supplementary material

Appendix

## Data Availability

The study data are available upon request from the division of biostatistics of the Uganda MoH and Makerere University Economic Policy Research Centre through the following contacts: emukooyo@gmail.com and ikasirye@eprcug.org, respectively.
