# Supplementary material for: Measuring health facility readiness and its effects on severe malaria outcomes in Uganda
Source: Sci Rep. 2018 Dec 18;8:17928. doi: 10.1038/s41598-018-36249-8 (PMC6298957; doi:10.1038/s41598-018-36249-8)
Supplement: Supplementary file 1 — Appendix [file 41598_2018_36249_MOESM1_ESM.pdf]

Measuring health facility readiness and its effects on severe malaria outcomes in Uganda

Julius Ssempiira, Ibrahim Kasirye, John Kissa, Betty Nambuusi, Eddie Mukooyo, Jimmy Opigo, Fredrick Makumbi, Simon Kasasa, Penelope Vounatsou

A1. Model formulation

A geostatistical negative binomial model [47] was fitted to assess the effect of the facility readiness index on malaria related deaths in HCIIIs or severe malaria cases in HCIIIs and HCIIs. Let Y_i be the cumulative count of malaria related deaths or severe malaria cases reported by health facility i during January – December 2013. Y_i is assumed to follow a negative binomial distribution, $Y_i \sim NB\ (p_i, r)$ where $p_i = {}^{r}/_{r\ +\mu_i}$ and r is the dispersion parameter of the distribution. We relate the predictors to the mean count μ_i of the malaria outcome reported at facility i via the log-linear regression equation, $log(\mu_i) = \log(N_i) + \boldsymbol{\beta}^T\boldsymbol{X} + \omega_i + \varphi_i$ where N_i is the offset which was considered to be the total number of severe malaria cases for the malaria deaths outcome and the total number of confirmed malaria cases for the severe malaria cases outcome. $\boldsymbol{X}$ are the predictors, that is, the facility readiness index and facility characteristics, and $\boldsymbol{\beta}$ is the vector of regression coefficients. ω_i are facility location random effects added in the model to account for spatial dependence in the rates of severe malaria morbidity/mortality. We assumed a Gaussian process on $\boldsymbol{\omega} = (\omega_1, \omega_2, \dots, \omega_k)^T$, that is, $\boldsymbol{\omega} \sim N(0, \sigma^2 R)$ where R is a correlation matrix, defined by an exponential parametric function of the distance d_{ij} between the locations of facilities i and j i.e. $R_{ij} = \exp(-d_{ij}\rho)$. The parameter σ^2 measures the spatial variation and ρ is a smoothing parameter that controls the rate of correlation decay with increasing distance. The range parameter, $\frac{3}{\rho}$ estimates the minimum distance beyond which spatial correlation is negligible. Non-spatial variation is estimated by the location random effects φ_i, which are assumed to be independent and normally distributed with mean 0 and variance σ_φ^2 , that is, $\varphi_i \sim N(0, \sigma_\varphi^2)$. Model fit and parameter estimation was performed using Bayesian formulation and Markov Chain Monte Carlo (MCMC) estimation. Model specification was completed by assigning prior distributions to model parameters. An inverse-gamma hyperprior was assigned to the

variance parameter σ_φ^2, a gamma distribution was adopted for the spatial smoothing parameter, and non-informative Gaussian distributions for the regression coefficients with mean 0 and variance 100. Model parameters were estimated using MCMC simulation, running a two-chain algorithm with a burn-in of 10,000 iterations followed by 200,000 iterations. Convergence was formally assessed by the Gelman and Rubin diagnostic [48], implemented in CODA.

A2. Multiple correspondence analysis

Let K denote the number of binary readiness indicators, N be the number of health facilities and $\boldsymbol{X}_{Nx(2*K)}$ denote the indicator matrix in which the facilities are displayed as rows and each indicator/tracer is represented by the inclusion of two columns $\boldsymbol{X}_{j_k}^k$, one per category of the tracer $k = 1, \dots, K,$ corresponding to its presence $(j_k = 1)$ or absence $(j_k = 0)$ from the facility. Let $\boldsymbol{P}$ be the matrix, $\boldsymbol{P} = \frac{1}{N*K}\boldsymbol{X}, \boldsymbol{r}$ and $\boldsymbol{c}$ the vectors of the row and column totals of $\boldsymbol{P}$, respectively, and $\boldsymbol{S}$ the matrix $\boldsymbol{S} = \boldsymbol{D}_r^{-\frac{1}{2}}(\boldsymbol{P} - \boldsymbol{rc}^T)\boldsymbol{D}_c^{-\frac{1}{2}}$ where $\boldsymbol{D}_r = diag\{\boldsymbol{r}\}$ and $\boldsymbol{D}_c = diag\{\boldsymbol{c}\}$. A readiness score F_i^a corresponding to health facility i and based on the ath factorial axis of MCA was defined by $F_i^a = \frac{1}{K}\sum_{k=1}^K \sum_{j_k \in \{0,1\}} W_{j_k}^{a,k} X_{j_k,i}^k$ where the weights $W_{j_k}^{a,k}$ are the corresponding column standard coordinates of the ath factorial axis, that is, are elements of the ath column of the matrix $\boldsymbol{D}_c^{-\frac{1}{2}}\boldsymbol{V}$ where $\boldsymbol{V}$ is the right singular vector of $\boldsymbol{S}$. The factorial score of the first axis is then defined by $F_i^1 = \frac{1}{K}\sum_{k=1}^K \sum_{j_k \in \{0,1\}} W_{j_k}^{1,k} X_{j_k,i}^k$. The variance explained by the ath factorial axis is given by the eigenvalues $\lambda_a = (\boldsymbol{D}_S^2)_{\boldsymbol{a}}$ where $\mathbf{D}_S$ is a diagonal matrix with the singular values of **S.**

A3. Construction of a composite readiness score

Following the approach proposed by Asselin (2009), we defined for each indicator k a discrimination measure Δ_k^a on each factorial axis a, $\Delta_k^a = \sum_{k\in\{0,1\}} \frac{n_{j_k}^k}{N} (W_{j_k}^{a,k})^2$ where $n_{j_k}^k$ is the absolute frequency of the j_kth category of indicator k. The average of the discrimination measures across the K indicators on the ath axis corresponds to the total variance explained by the axis, that is, $\lambda_a = \frac{1}{K} \sum_{k=1}^{K} \Delta_k^a$.

For each factorial axis, we split the indicators in two groups, each satisfying the Axis Ordering Consistency condition (AOC) in one of the two axis orientations, i.e. positive (G_1) or negative (G_2). We then calculated the total variance explained by each group in the axis, that is, $\Delta_{G_j}^a = \sum_{k\in G_j} \Delta_k^a$ where $j = 1,2$ and retained on the axis the group of indicators explaining more variation than a threshold T_a which was taken to be 50% of the variance explained by the axis, that is, $\mathrm{T}_a = 0.5 * K * \lambda_a$. The groups of indicators retained on the axes, are overlapping and an indicator can be retained on several axes. We removed intersections by selecting the factorial axis with the highest discrimination measure for that indicator among all axes. We defined the composite readiness score $F_i = \frac{1}{K} \sum_{k=1}^{K} \sum_{j_k\in\{0,1\}} \sum_{a=1}^{L} \delta(k-a) W_{j_k}^{a,k} X_{j_k,i}^k$ where L is the number of factorial axes used in the composite score and $\delta(k-a)$ is the Dirac delta function which takes the value 1 when the kth indicator is retained on the ath factorial axis and 0 otherwise, that is $\delta(k-a) = 1$ if $k = a$ and $\delta(k-a) = 0$ if $k \neq a$. To improve interpretation of the score we translated the weights so that the absence category $(j_k = 0)$ of the k indicator receives a zero and the presence one $(j_k = 1)$ receives a strictly positive weight representing the gain in the readiness increase measured by the axis a when a facility i acquires the kth tracer. Therefore the $W_{j_k}^{a,k}$ in F_i was replaced by $W_{j_k}^{+a,k}$ where $W_0^{+a,k} = 0$ and $W_1^{+a,k}$=$W_1^{+a,k} - W_0^{a,k}$.

A4. Geostatistical variable selection

To identify the most important readiness indicators related to malaria deaths and severe malaria cases, Bayesian geostatistical variable selection was implemented using stochastic search and adopting spike and slab prior distributions for the regression coefficients [27]. For every readiness indicator X_k a Bernoulli variable γ_k was introduced with Bernoulli probability π_k corresponding to the inclusion of X_k in the model. For the coefficient β_k, we assume a prior distribution which is a mixture of non-informative normal distributions, $\beta_k \sim \delta(\gamma_{k-1}) N(0, \tau_k^2) + \left(1 - \delta(\gamma_{k-1})\right) N(0, \vartheta_0 \tau_k^2)$ where $\delta(.)$ is the Dirac delta function. Therefore, in case X_k is included in the model, the prior distribution β_k has a non-informative prior distribution $\beta_k \sim N(0, \tau_k^2)$ and in case X_k is excluded, $\beta_k \sim N(0, \vartheta_0 \tau_k^2)$ where $\vartheta_0 = 10^{-5}$ is a very small number shrinking the variance to zero i.e. spike component of the prior. We have adopted a $Beta(1,1)$ hyperprior for π_k and an inverse gamma prior for the variance τ_k^2 with mean 1 and variance 10.

Table A1: Frequency distribution and chi-square test results of general service and malaria-specific readiness indicators compared by level and facility characteristics

Indicator	Total (N=201) n (%)	Facility level			Managing authority			Location			Distance to district headquarters		
		HCIIIs N=105	HCIIs N=96	P-value	Public N=146	Private N=55	P-value	Rural N=166	Urban N=35	P-value	0-10 km N=52	>10 km N=149	P-value
		n(%)	n(%)		n(%)	n(%)		n(%)	n(%)		n(%)	n(%)	
**Basic amenities**	**3 (1.5)**	**3 (2.9)**	**0 (0.0)**	**0.095**	**1 (0.7)**	**2 (3.6)**	**0.124**	**1 (0.6)**	**2 (5.7)**	***0.023***	**2 (3.9)**	**1 (0.7)**	**0.104**
Uninterrupted power supply	77 (38.3)	45 (42.9)	32 (33.3)	0.165	56 (36.4)	21 (38.2)	0.982	67 (40.4)	10 (28.6)	0.192	21 (40.4)	56 (37.6)	0.721
Improved water source inside or within source of facility	58 (28.9)	37 (35.2)	21 (21.9)	*0.037*	37 (25.3)	21 (38.2)	0.073	46 (27.7)	12 (34.3)	0.435	19 (36.5)	39 (26.2)	0.156
Access to adequate sanitation facilities for clients	182 (90.6)	94 (89.5)	88 (91.7)	0.604	135 (92.5)	47 (85.5)	0.130	152 (91.6)	30 (85.7)	0.282	45 (86.5)	137 (92.0)	0.251
Communication equipment (phone or short wave radio)	28 (13.9)	22 (21.0)	6 (6.3)	*0.003*	10 (6.9)	18 (32.7)	*<0.0001*	14 (4.8)	14 (40.0)	*<0.0001*	11 (21.2)	17 (11.4)	0.081
Access to computer with email/internet access	29 (14.4)	21 (20.0)	8 (8.3)	*0.019*	12 (8.2)	17 (30.9)	*<0.0001*	16 (9.6)	13 (37.1)	*<0.0001*	11 (21.2)	18 (12.1)	0.109
Emergency transportation	21 (10.5)	16 (15.2)	5 (5.2)	*0.020*	4 (2.7)	17 (30.9)	*<0.0001*	12 (7.2)	9 (25.7)	*0.001*	7 (13.5)	14 (9.4)	0.409
**Basic equipment**	**101 (50.3)**	**63 (60.0)**	**38 (39.6)**	**0.004**	**63 (43.2)**	**38 (69.1)**	***0.001***	**77 (46.4)**	**24 (68.6)**	***0.017***	**26 (50.0)**	**75 (50.3)**	**0.967**
Adult scale	157 (78.1)	87 (82.9)	70 (72.9)	0.089	106 (72.6)	51 (92.7)	*0.002*	126 (75.9)	31 (88.6)	0.100	36 (69.2)	121 (81.2)	0.072
Child scale	159 (79.1)	89 (84.8)	70 (72.9)	*0.039*	116 (79.5)	43 (78.2)	0.843	132 (79.5)	27 (77.1)	0.753	38 (73.1)	121 (81.2)	0.214
Thermometer	163 (81.1)	88 (83.8)	75 (78.1)	0.304	112 (76.7)	51 (92.7)	*0.010*	129 (77.7)	34 (97.1)	*0.008*	43 (82.7)	120 (80.5)	0.733
Stethoscope	178 (88.6)	98 (93.3)	80 (83.3)	*0.026*	124 (84.9)	54 (98.2)	*0.009*	144 (86.8)	34 (97.1)	0.079	47 (90.4)	131 (87.9)	0.631
Blood pressure apparatus	168 (83.6)	91 (86.7)	77 (80.2)	0.217	119 (81.5)	49 (89.1)	0.196	137 (82.5)	31 (88.6)	0.381	43 (82.7)	125 (83.9)	0.841
**Standard precautions for infection prevention**	**9 (4.9)**	**5 (4.8)**	**4 (4.2)**	**0.838**	**3 (2.1)**	**6 (10.9)**	***0.007***	**4 (2.4)**	**5 (14.3)**	***0.002***	**3 (5.8)**	**6 (4.0)**	**0.601**
Sterilization equipment	36 (17.9)	29 (27.6)	7 (7.3)	*<0.0001*	18 (12.3)	18 (32.7)	*0.001*	25 (15.1)	11 (31.4)	*0.022*	9 (17.3)	27 (18.1)	0.895
Appropriate storage	194 (96.5)	101 (96.2)	93 (96.9)	0.791	142 (97.3)	52 (94.6)	0.349	161 (97.0)	33 (94.3)	0.428	49 (94.2)	145 (97.3)	0.296

Indicator	Total (N=201) n (%)	Facility level			Managing authority			Location			Distance to district headquarters		
		HCIIIs N=105	HCIIs N=96	P-value	Public N=146	Private N=55	P-value	Rural N=166	Urban N=35	P-value	0-10 km N=52	>10 km N=149	P-value
		n(%)	n(%)		n(%)	n(%)		n(%)	n(%)		n(%)	n(%)	
of sharps waste													
Safe final disposal of sharps	25 (12.4)	15 (14.3)	10 (10.4)	0.406	12 (8.2)	13 (23.6)	*0.003*	20 (12.1)	5 (14.3)	0.715	8 (15.4)	17 (11.4)	0.455
Disposable syringes with disposable needles	194 (96.6)	101 (96.2)	93 (96.9)	0.791	141 (96.6)	53 (96.4)	0.942	159 (95.8)	35 (100.0)	0.216	49 (94.2)	145 (97.3)	0.296
Disposable gloves	192 (95.5)	98 (93.3)	94 (97.9)	0.117	138 (94.5)	54 (98.2)	0.263	159 (95.8)	33 (94.3)	0.697	51 (98.1)	141 (94.6)	0.301
**Diagnostic capacity**	**40 (19.9)**	**34 (32.4)**	**6 (6.3)**	***<0.0001***	**24 (16.4)**	**16 (29.1)**	***0.045***	**28 (16.9)**	**12 (34.3)**	***0.019***	**14 (26.6)**	**26 (17.5)**	**0.141**
Malaria RDTs	155 (77.1)	83 (79.1)	72 (75.0)	0.495	118 (80.8)	37 (67.3)	*0.041*	134 (80.7)	21 (60.0)	*0.008*	38 (73.1)	117 (78.5)	0.421
Blood glucose	64 (31.8)	52 (49.5)	12 (12.5)	*<0.0001*	34 (23.3)	30 (54.6)	*<0.0001*	43 (25.9)	21 (60.0)	*<0.0001*	20 (38.5)	44 (29.5)	0.234
HIV diagnostic capacity	126 (62.7)	89 (84.8)	37 (38.5)	*<0.0001*	87 (59.6)	39 (70.9)	0.139	98 (59.0)	28 (80.0)	*0.020*	38 (73.1)	88 (59.1)	0.072
Urine dipstick	88 (43.8)	74 (70.5)	14 (14.6)	*<0.0001*	56 (38.4)	32 (58.2)	*0.012*	67 (40.4)	21 (60.0)	*0.033*	27 (51.9)	61 (40.9)	0.169
**Essential medicines**	**5 (2.5)**	**5 (4.8)**	**0 (0.0)**	***0.030***	**1 (0.7)**	**4 (7.3)**	***0.008***	**4 (2.41)**	**1 (2.86)**	**0.877**	**0 (0)**	**5 (3.7)**	**0.181**
Amoxicillin syrup/suspension or dispersible tablet	41 (20.4)	24 (22.9)	17 (17.7)	0.366	6 (4.1)	35 (63.6)	*<0.0001*	24 (14.5)	17 (48.6)	*<0.0001*	15 (28.9)	26 (17.5)	0.079
Ampicillin powder for injection	79 (39.3)	72 (68.6)	7 (7.3)	*<0.0001*	61 (41.8)	18 (32.7)	0.241	61 (36.8)	18 (51.4)	0.106	24 (46.2)	55 (36.9)	0.240
Ceftriaxone injection	101 (50.3)	41 (39.1)	60 (62.5)	*0.001*	64 (43.8)	37 (67.3)	*0.003*	81 (48.8)	20 (57.1)	0.369	26 (50.0)	75 (50.3)	0.967
Gentamicin injection	73 (36.3)	52 (49.5)	21 (21.9)	*<0.0001*	32 (21.9)	41 (74.6)	*<0.0001*	53 (31.9)	20 (57.1)	*0.005*	16 (30.8)	57 (38.3)	0.334
Magnesium sulphate injectable	63 (31.3)	58 (55.2)	5 (5.2)	*<0.0001*	51 (34.9)	12 (21.8)	0.074	54 (32.5)	9 (25.7)	0.430	15 (28.9)	48 (32.2)	0.652
Oral rehydration solution	161 (80.1)	87 (82.9)	74 (77.1)	0.306	116 (79.5)	45 (81.8)	0.708	129 (77.7)	32 (91.4)	0.065	42 (80.8)	119 (79.9)	0.888
Oxytocin injection	63 (31.3)	58 (55.2)	5 (5.2)	*<0.0001*	51 (34.9)	12 (21.8)	0.074	54 (32.5)	9 (25.7)	0.430	15 (28.9)	48 (32.2)	0.652
Zinc sulphate tablets, dispersible tablets or syrup	141 (70.2)	77 (73.3)	64 (66.7)	0.302	111 (76.0)	30 (54.6)	0.003	118 (71.1)	23 (65.7)	0.528	38 (73.1)	103 (69.1)	0.592

Indicator	Total (N=201) n (%)	Facility level			Managing authority			Location			Distance to district headquarters		
		HCIIIs N=105	HCIIs N=96	P-value	Public N=146	Private N=55	P-value	Rural N=166	Urban N=35	P-value	0-10 km N=52	>10 km N=149	P-value
		n(%)	n(%)		n(%)	n(%)		n(%)	n(%)		n(%)	n(%)	
**Malaria service**	**53 (26.4)**	**45 (42.9)**	**8 (8.3)**	***<0.0001***	**32 (21.9)**	**21 (38.2)**	***0.020***	**44 (26.5)**	**9 (25.7)**	**0.923**	**13 (25.0)**	**40 (26.9)**	**0.795**
Thermometer	163 (81.1)	88 (83.8)	75 (78.1)	0.304	112 (76.7)	51 (92.7)	*0.010*	129 (77.7)	34 (97.1)	*0.008*	43 (82.7)	120 (80.5)	0.733
Malaria diagnosis by RDT	155 (77.1)	83 (79.1)	72 (75.0)	0.495	118 (80.8)	37 (67.3)	0.041	134 (80.7)	21 (60.0)	*0.008*	38 (73.1)	117 (78.5)	0.421
Malaria diagnosis by microscopy	97 (48.3)	81 (77.1)	16 (16.7)	*<0.0001*	59 (40.4)	38 (69.1)	*<0.0001*	72 (43.4)	25 (71.4)	*0.003*	27 (51.9)	70 (47.0)	0.539
Malaria treatment (ACTs)	174 (86.6)	88 (83.8)	86 (89.6)	0.231	127 (87.0)	47 (85.5)	0.776	146 (88.0)	28 (80.0)	0.210	43 (82.7)	131 (87.9)	0.341
Intermittent preventive treatment (Fancidar)	171 (85.1)	94 (89.5)	77 (80.2)	0.064	127 (87.0)	44 (80.0)	0.215	144 (86.8)	27 (77.1)	0.147	44 (84.6)	127 (85.2)	0.914
Artesunate	7 (3.5)	5 (4.8)	2 (2.1)	0.301	0 (0)	7 (12.7)	*<0.0001*	4 (2.4)	3 (8.6)	0.071	2 (3.9)	5 (3.4)	0.868

Bold: Domain readiness indicators

Italics: Significant values

Table A2: Selection of factorial axes included in the composite score for HCIIIs

Indicators	Discrimination measures							Selected axis	Weights[b] $W_1^{+\alpha,k}$
	Factorial axes[a]								
	1	2	3	4	5	6	7		
Improved water source inside or within source	0.075	0.133	0.051	0.126	0.001	**0.435**	0.158	6	4912
Adult scale	0.003	**0.431**	0.136	0.056	0.003	0.014	0.057	2	4656
Disposable gloves	0.047	0.004	0.089	**0.500**	0.034	0.178	0.090	4	8922
Malaria diagnostic capacity	0.045	0.006	0.187	0.001	**0.569**	0.007	0.010	5	5888
Ampicillin powder for injection	**0.342**	0.020	0.166	0.001	0.021	0.002	0.046	1	2628
Ceftriaxone injection	0.001	**0.356**	0.067	0.157	0.192	0.003	0.001	2	3267
Gentamicin injection	0.012	**0.516**	0.021	0.028	0.104	0.038	0.006	2	3839
Magnesium sulphate injectable	**0.811**	0.006	0.135	0.007	0.001	0.003	0.004	1	3777
Oxytocin injection	**0.811**	0.006	0.135	0.007	0.001	0.003	0.004	1	3777
Zinc sulphate tablets	0.188	0.003	0.173	0.082	0.158	0.001	**0.273**	7	4287
Microscopy	**0.194**	0.061	0.082	0.146	0.005	0.184	0.187	1	2188
**Variance threshold (T_a)**	1.265	0.769	0.622	0.556	0.545	0.435	0.418		
**Variation explained ($\Delta_{G_1}^{a}$)**	2.391	1.384	0.478	0.680	0.392	0.005	0.551		
**Variation explained ($\Delta_{G_2}^{a}$)**	0.138	0.158	0.764	0.431	0.697	0.863	0.285		
**Variation explained after eliminating intersection axis**	2.158	1.303	0.000	0.500	0.569	0.435	0.273		

[a]Shaded grey cells (Group 1-positive orientation); Unshaded (Group 2 -negative orientation)
[b]Weights were multiplied by 1000

Table A2: Selection of factorial axes included in the composite score for HCIIs

Indicator	Discrimination measures					Selected axis	Weights[b] $W_1^{+\alpha,k}$
	Factorial axes[a]						
	**1**	**2**	**3**	**4**	**5**		
Emergency transportation	**0.604**	0.000	0.026	0.001	0.025	1	6779
Child scale	0.045	**0.404**	0.051	0.020	0.295	2	3969
Appropriate storage of sharps waste	0.010	0.261	0.126	0.214	**0.309**	5	10954
Single use standard disposable or auto-disable syringes	0.027	0.011	**0.519**	0.114	0.062	3	12156
Disposable gloves	0.001	0.285	0.048	**0.525**	0.039	4	16125
Glucometer	**0.641**	0.022	0.001	0.055	0.000	1	4695
Amoxicillin syrup/suspension or dispersible tablet	**0.327**	0.190	0.024	0.001	0.047	1	2904
Thermometer	0.090	0.090	**0.338**	0.038	0.071	3	4129
Microscopy	**0.547**	0.020	0.015	0.017	0.000	1	3848
Artesunate	**0.368**	0.017	0.013	0.004	0.001	1	8239
**Variance threshold** (T_a)	1.330	0.650	0.580	0.495	0.425		
**Variation explained** ($\Delta_{G_1}^{a}$)	2.659	0.511	0.280	0.928	0.421		
**Variation explained** ($\Delta_{G_2}^{a}$)	0.001	0.788	0.881	0.061	0.428		
**Variation explained after eliminating intersection axis**	2.487	0.404	0.857	0.525	0.309		

[a]Shaded grey cells (Group 1-positive orientation); Unshaded (Group 2 -negative orientation)
[b]Weights were multiplied by 1000

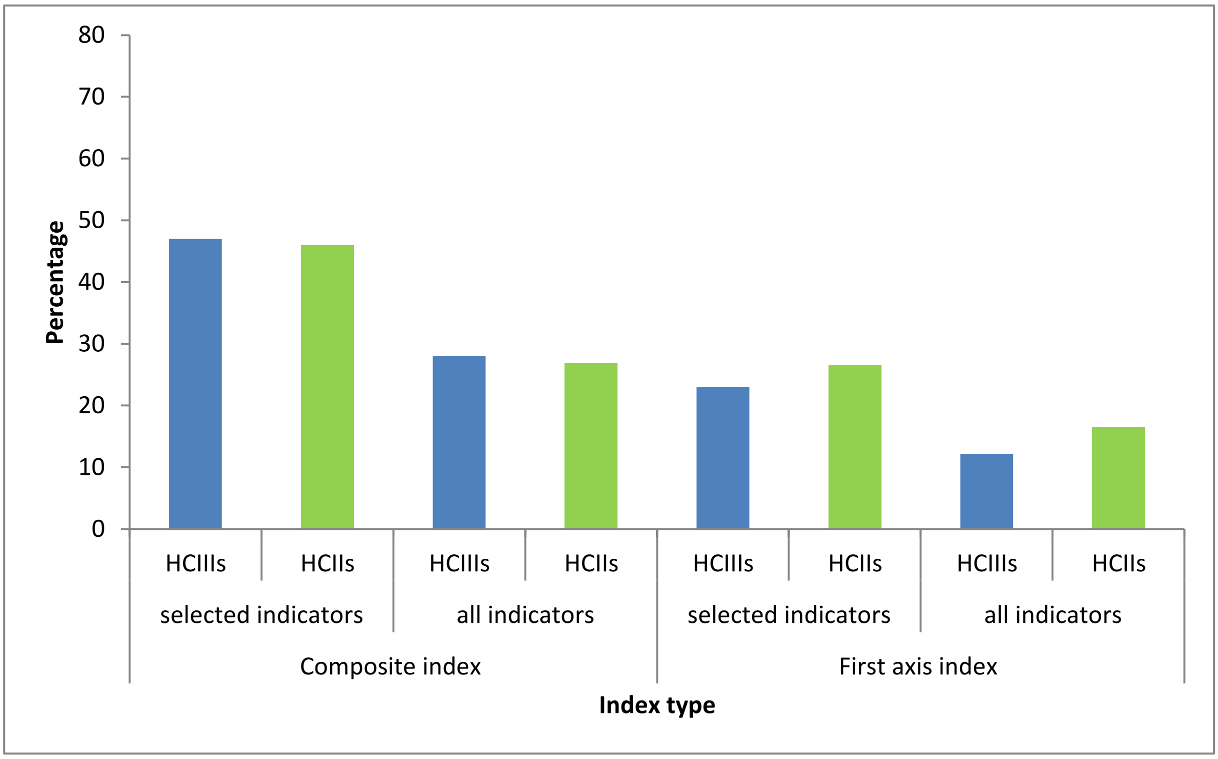

Fig. A1: Proportion of variation explained by the composite score and the score based on the first factorial axis for HCIIIs (blue) and HCIIs (green)

(a) (b)

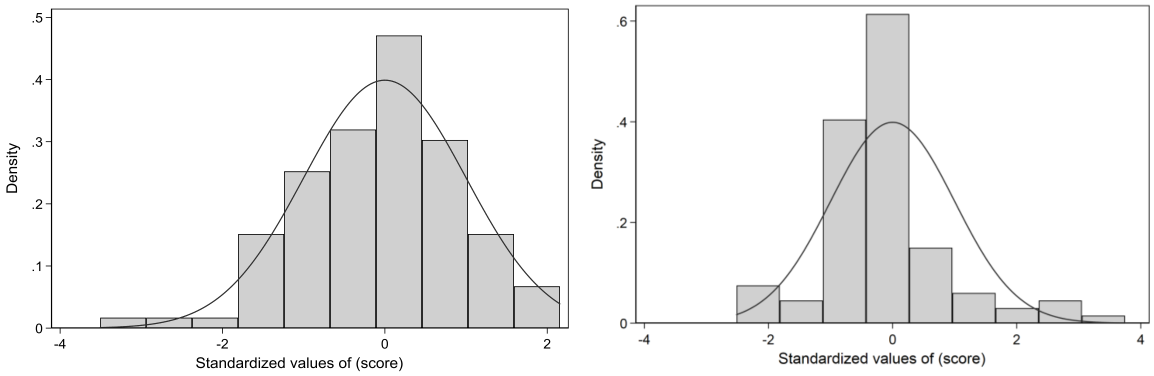

Fig. A2: Distribution of facility readiness score; HCIIIs (left) and HCIIs (right)

Table A3: Posterior estimates of the effects of composite facility readiness index on severe malaria outcomes based on all indicators

Characteristic	HCIIIs		HCIIs
	Malaria deaths	Severe malaria cases	Severe malaria cases
	IRR (95%BCI)[1]	IRR (95%BCI)	IRR (95%BCI)
**Readiness index**			
Low	1	1	1
Medium	1.96 (0.68, 2.54)	0.29 (0.21, 0.44)*	1.33 (0.58, 1.42)
High	0.65 (0.31, 1.20)	0.44 (0.35, 0.57)*	1.53 (0.91, 1.72)
**Location**			
Rural	1	1	1
Urban	0.62 (0.22, 0.99)*	1.37 (1.13, 2.02)*	2.48 (1.20, 4.85)*
**Ownership**			
Government	1	1	1
Private	1.35 (0.83, 1.71)	9.36 (7.00, 11.64)*	3.23 (1.75, 3.93)*
**Distance to district headquarters**			
<=10km	1	1	1
>10km	0.44 (0.19, 0.86)*	1.27 (0.56, 1.58)	3.98 (3.01, 6.12)*
**Spatial parameters**			
Spatial variance	0.50 (0.37, 0.60)	0.61 (0.49, 0.99)	0.68 (0.54, 0.87)
Range (km)	5.47 (2.77, 16.64)	4.26 (2.73, 13.21)	35.51 (4.65, 70.31)

**statistically important effect; [1]IRR: Incidence Rate Ratio*
